# Trait-based community assembly of epiphytic diatoms in saline astatic ponds: a test of the stress-dominance hypothesis

**DOI:** 10.1038/s41598-019-52304-4

**Published:** 2019-10-31

**Authors:** Éva Ács, Angéla Földi, Csaba Ferenc Vad, Zsuzsa Trábert, Keve Tihamér Kiss, Mónika Duleba, Gábor Borics, István Grigorszky, Zoltán Botta-Dukát

**Affiliations:** 1 MTA Centre for Ecological Research, Danube Research Institute, Karolina út 29, H-1113 Budapest, Hungary; 2 National University of Public Service, Faculty of Water Sciences, 6500 Baja, Bajcsy-Zsilinszky utca 12-14, Hungary; 3 Doctoral School of Environmental Sciences, Eötvös Lóránd University, Budapest, Hungary; 4 Wasser Cluster Lunz, Dr. Carl Kupelwieser Promenade 5, A-3293 Lunz am See, Austria; 5 MTA Centre for Ecological Research, Sustainable Ecosystems Group, Klebelsberg Kuno u. 3, H-8237 Tihany, Hungary; 6 University of Debrecen, Department of Hydrobiology, Egyetem tér 1., H-4032 Debrecen, Hungary; 7 MTA Centre for Ecological Research, Institute of Ecology and Botany, Alkotmány u. 2-4., H-2163 Vácrátót, Hungary

**Keywords:** Freshwater ecology, Community ecology

## Abstract

The stress dominance hypothesis (SDH) postulates that strong environmental gradients drive trait convergence in communities over limiting similarity. Previous studies, conducted mostly with terrestrial plant communities, found controversial evidence for this prediction. We provide here the first test for SDH for epiphytic diatoms. We studied community assembly in diatom communities of astatic ponds. These water bodies serve as a good model system for testing SDH because they exhibit stress gradients of various environmental factors. Functional diversity of diatom communities was assessed based on four traits: (1) combined trait reflecting the trade-off between stress tolerance and competitive dominance, (2) cell size, (3) oxygen requirement and (4) N-uptake strategy. According to our results, salinity, pH and the width of the macrophyte belt appeared as significant predictors of the trait convergence/divergence patterns presumably acting through influencing the availability of carbon dioxide and turbidity. Lower trait diversity was found in turbid, more saline and more alkaline ponds and functional diversity was higher in transparent, less saline and less alkaline ponds. Overall, our results supported the stress dominance hypothesis. In habitats representing increased environmental stress, environmental filtering was the most important community assembly rule, while limiting similarity became dominant under more favourable conditions.

## Introduction

Understanding the major processes driving the assembly of local communities from a regional species pool is a central topic of community ecology^1^. These processes operate as a series of filters^1,2^ including dispersal limitation, environmental suitability, and interactions between co-occurring species. The dispersal potential of small-bodied organisms, such as bacteria and protists is considered to be enormous, which suggests an insignificant role of dispersal limitation on small spatial scales^3^, albeit exceptions can occur^4^. Community assembly studies often focus on the other two filters, which are: environmental suitability and interactions among competing species. Trait-based approaches are timely tools for building a mechanistic framework for community assembly and are increasingly used, especially in terrestrial plant communities^5^. They are less considered in aquatic primary producers, though their huge phylogenetic diversity and related trait-differences make them highly suitable objects for applying these concepts^6–9^.

Environmental filtering *sensu stricto* determines which species of the regional species pool can establish and persist in local populations in a given habitat^10^ in the absence of biotic interactions. Filtering, however, is deterministic only in the sense that the environment determines the probability of the occurrence, and species with a low probability of occurrence can be regarded as filtered out^1^. Abiotic conditions influence not only the probability of presence but also the probability of reaching a high abundance. Since both effects on presence and abundance lead to trait convergence (i.e. a lower functional diversity than would be expected in random trait assembly)^11,12^, here we do not distinguish them and refer to both as environmental filtering. According to the niche theory^13,14^, for robust co-existence species have to differ in their population regulation. This limiting similarity causes divergence in traits related to resource use, defence against natural enemies and other processes regulating population growth^15^. Both environmental filtering and limiting similarity may influence community assembly simultaneously; therefore, both trait convergence and trait divergence may occur in a given habitat depending on the type of the functional traits studied^16–18^.

Testing the hypotheses of how ecological processes vary along environmental gradients may reduce the likelihood of drawing incorrect conclusions from analyses of functional diversity^19^. Productivity and various stress gradients are those along which the functional properties of assemblages are most frequently studied. Several theories have been proposed to predict how trait convergence or divergence vary along these gradients^20–23^.

The stress-dominance hypothesis (SDH) predicts that in a harsh environment habitat filtering is the major driver of community composition resulting in strong trait convergence, while limiting similarity is more important in less stressful habitats, resulting in trait divergence^20,23,24^. Empirical evidence for this theory from terrestrial plant or phytoplankton communities is controversial. Mason *et al*.^25^ found evidence for the increasing importance of limiting similarity in more productive environments. Other studies found the opposite, a transition in the relative contribution of trait divergence to trait convergence with increasing productivity e.g. ^17,26,27^, which contradicts the postulate of the stress-dominance hypothesis. At the same time, these findings support Grime’s^21^ predictions that higher productivity should lead to trait convergence because increasing competition excludes species with traits associated with weaker competitive ability. Combining the two hypotheses, Navas & Violle^22^ argued that trait convergence could be expected at both ends of the productivity gradient, and trait divergence at medium productivity. Lhotsky *et al*.^18^ showed that the sign of changes along an environmental gradient might depend on the role of the studied traits: in their analysis, the pattern of the vegetative traits supported the stress-dominance hypothesis, while the pattern of the seed weight diversity contradicted it. In phytoplankton, Kruk *et al*.^28^ found evidence that on a global scale higher productivity and environmental stability lead to a smaller variance in traits.

Epiphytic diatoms are ideal objects for testing concepts on the role of environmental filtering and limiting similarity because they are small-bodied passive dispersers, frequent in aquatic environments, form an extremely diverse phylum (with more than 100,000 taxa) and well-developed methods exist for their routine microscopical identification. They have long been used as bioindicators^29^ because they are highly sensitive to changes in environmental conditions (e.g. nutrient concentration, salinity, pH, turbidity)^30^. Based on their indication ability, benthic diatoms are widely used in ecological status assessment both in riverine and lacustrine environments^31,32^. To the best of our knowledge, the stress-dominance hypothesis has not yet been tested in benthic diatom communities.

As a model system for studying the assembly of epiphytic diatom communities, we have chosen a dense cluster of saline bomb crater ponds^33^. These ponds represent ideal sites for testing theories of community assembly along stress gradients because they are restricted in space and similar in morphology, but exhibit gradients of several environmental variables.

The salinity gradient constitutes a major factor structuring aquatic communities^34^. In contrast to the productivity-diversity relationship which may show different (hump-shaped, negative, positive) patterns^35–37^, the species richness of zooplankton in inland saline waters shows a monotonous decline with increasing salinity^38^. Therefore, one can expect that the relative importance of environmental filtering over limiting similarity increases with increasing salinity.

In trait-based studies of benthic (including epiphytic, epilithic, etc.) diatoms^39^, beyond the frequently used ecological guilds^40,78^ and cell size^41^, oxygen requirement^30^ and N-uptake strategy^30^ could also be relevant traits.

We hypothesized that in the studied ponds the diatom species composition is predominantly determined by environmental filtering while limiting similarity has less importance in community assembly. Our aims were 1) to test the SDH in epiphytic diatom communities in sodic bomb crater ponds, which are astatic ponds with multiple stress gradients (including salinity, turbidity, pH); and to reveal 2) how the relative contribution of trait convergence/divergence to community assembly depends on environmental conditions.

## Results

All ponds were alkaline, and their physical and chemical properties exhibited considerable variation (see Supplementary Table S1). Significant correlations were found between continuous environmental variables, mainly between salinity, pH, N:P ratio and TP (see Supplementary Table S2). Salinity and pH were strongly correlated (Spearman’s rho = 0.74, p = 3.6*10^−9^; see Supplementary Fig. S1), and both were high when the macrophyte belt was absent and showed a decreasing trend with the increasing width of the macrophyte belt (see Supplementary Fig. S2).

Altogether, 80 diatom taxa, representing 33 genera, were identified in the ponds. The genus *Nitzschia* was represented by the greatest number of species (16), followed by the genus *Gomphonema* (9). Mean species richness was 13.7. Species richness did not change considerably along the salinity gradient (see Supplementary Fig. S3). According to the Chi-square tests, traits were only weakly associated with each other (see Supplementary Tables S3–S8).

**Figure 1:**
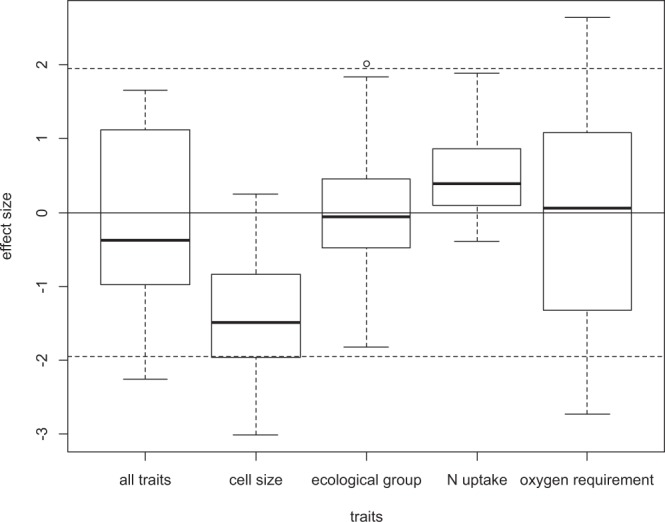
Box-plots of the effect sizes of each pond. A positive effect size indicates trait divergence, while a negative value indicates trait convergence. The threshold of the significant departure from zero at the 5% significance level is marked with dashed lines. Although most of the observed values are between these dashed lines, and the individual tests are therefore non-significant, several non-significant departures with the same direction may indicate significant departures from randomness at the meta-community level.

Considering the overall trait dispersion patterns (i.e. the effect size for all of the studied ponds irrespective of the environmental variables), trait convergence was dominant (most of the effect size values are negative) for cell size, while trait divergence was dominant for nitrogen uptake (most of the effect size values are positive). For these two traits the overall mean effect size departs significantly from zero: t = −11.583, p = 4.3*10^−15^, and t = 6.1156, p = 2.1*10^−7^, respectively. Both types of departure from randomness occurred for the ecological group, and for oxygen requirement, and also when all of four traits were used to calculate functional diversity (Fig. 1). In these cases mean values do not significantly depart from zero (t = 0.95942, p = 0.3425 for ecological group; and t = −0.0655, p = 0.9481 for oxygen requirement, and t = −0.90256, p-value = 0.3716 for all traits together).

**Figure 2:**
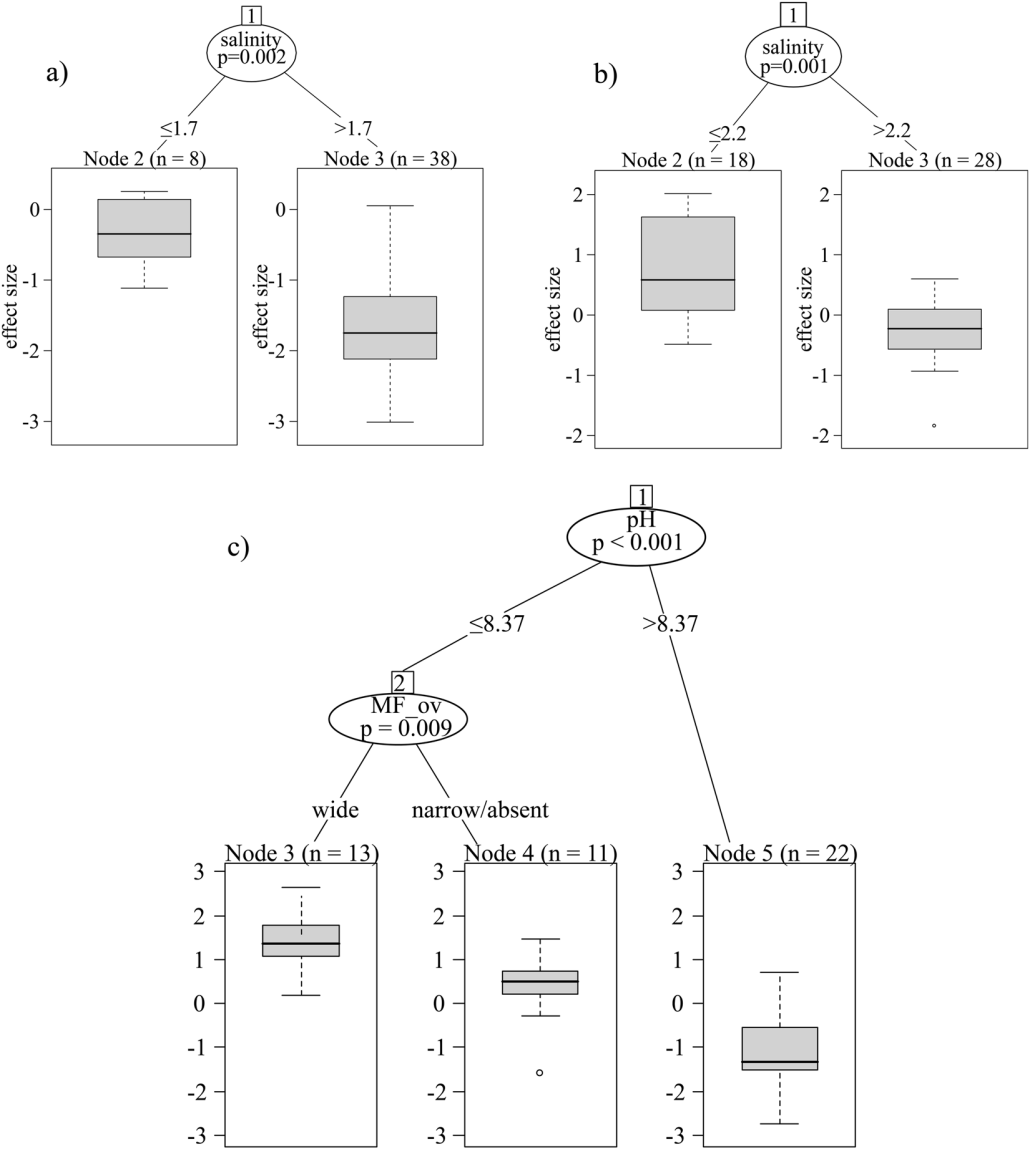
Dependence of effect sizes on environmental variables illustrated by the fitted conditional inference trees; (**a**) cell size, (**b**) ecological group, (**c**) oxygen requirement traits. n refers to the number of ponds belonging to the category. MP_belt: macrophyte belt.

**Figure 3:**
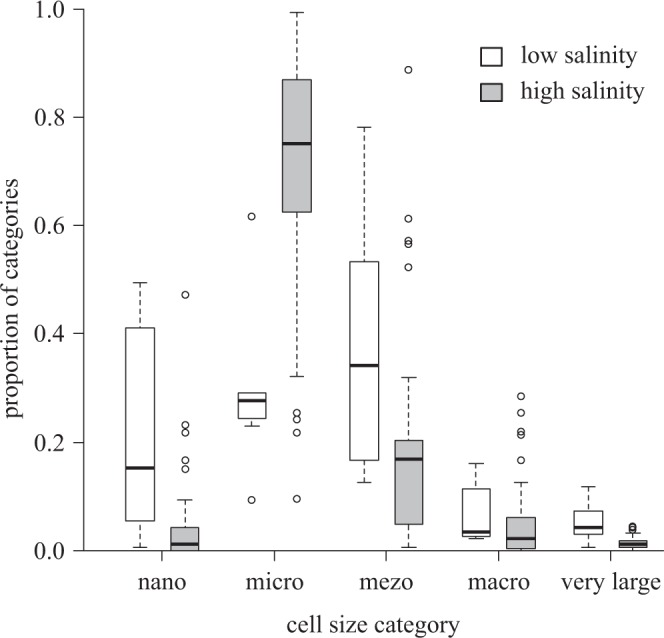
Proportion of cell size categories in ponds with low (white boxes) and high (grey boxes) salinity. Salinity categories correspond to two groups in Fig. 2a, i.e. high salinity here refers to values above 1.7 mg L^−1^. According to the Wilcoxon rank-sum test, the difference between the two groups is significant (p < 2.2*10^−16^) for all cell size categories.

Fitting a decision tree revealed that trait convergence in cell size was weak in low-salinity ponds (i.e. effect sizes are low in absolute values), but became strong (indicated by more negative effect sizes) if salinity exceeded 1.7 g L^−1^ (Fig. 2a). Macro- and very large diatoms did not reach high relative abundance, irrespective of environmental conditions (see Supplementary Table S9, Fig. 3). Low-salinity ponds (i.e. below 1.7 g L^−1^) were co-dominated by nano-, micro- and meso-sized diatoms; at high salinity (above 1.7 g L^−1^) the micro-sized group became the sole dominant group (Fig. 3).

**Figure 4:**
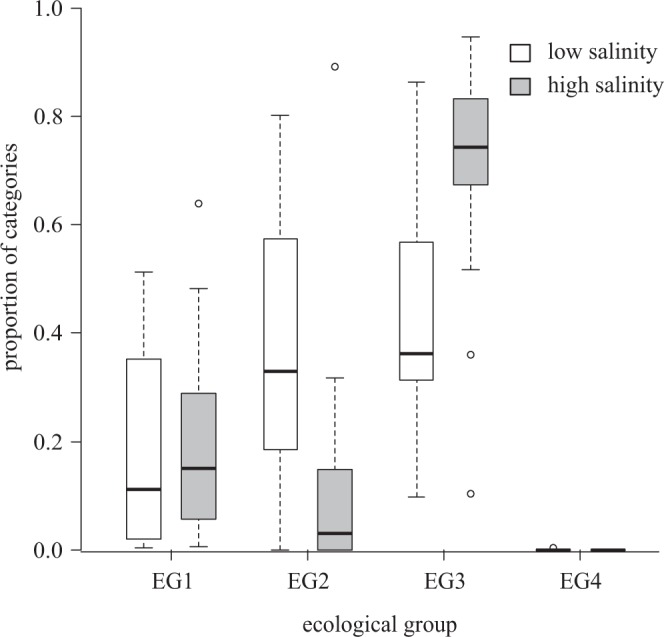
Proportion of ecological group categories in ponds with low (white boxes) and high (grey boxes) salinity. Salinity categories correspond to two groups in Fig. 2b, i.e. high salinity here refer to values above 2.2 mg L^−1^. According to the Wilcoxon rank-sum test, the difference between two groups is significant (p < 2.2*10^−16^) for all ecological group categories.

The functional diversity of the ecological group (EG) was higher than a random expectation (trait divergence) in ponds with salinity below 2.2 g L^−1^, while it hardly departed from a random expectation if salinity exceeded this threshold (Fig. 2b). The relative abundance of diatoms with EG4 was low in all ponds (see Supplementary Table S9, Fig. 4), while the relative abundance of the other three EG categories varied considerably, depending on salinity (Fig. 4). At low salinity (i.e. below 2.2 g L^−1^) all of the three EG categories could reach a high relative abundance; at high salinity (i.e. above 2.2 g L^−1^) diatoms with EG3 became dominant.

Regarding the N-uptake strategy, trait divergence was observed in almost all ponds, irrespective of environmental conditions.

Analysing the oxygen requirement, we found trait convergence in ponds with high pH (>8.37). Note that the macrophyte belt in these ponds was narrow or completely absent and the total suspended matter content was high. Effect sizes were close to zero in ponds with lower pH (<8.37) and with a narrow or absent macrophyte belt, while in ponds with a wide macrophyte belt and lower pH, effect sizes indicated trait divergence (Fig. 2c).

**Figure 5:**
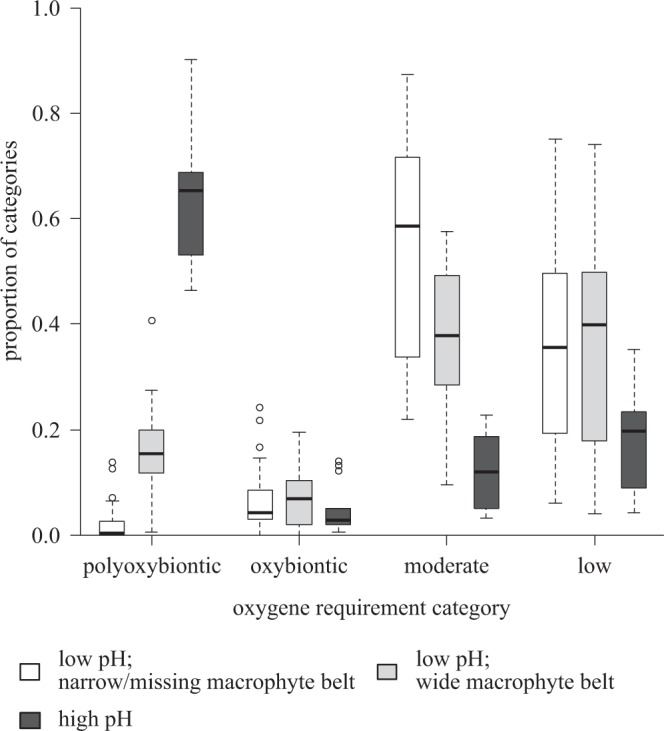
Proportion of oxygen requirement categories in ponds with high (white boxes) and low pH and a narrow/missing (light grey boxes) or wide macrophyte belt (dark grey boxes). The tree categories correspond to tree groups in Fig. 2c (i.e. high pH here refer to values above 8.37). According to the Kruskal-Wallis, there are significant differences among groups in the relative abundance of poly-oxybiont species (p = 1.842*10^−8^), and species with moderate (p = 1.608*10^−6^) and low (p = 0.04659) oxygen demand, while the relative abundance of oxybiont species varies significantly among groups (p = 0.685). Significant differences are indicated by different letters above the boxes.

The relative abundance of the oxybiontic species group varied in a narrow range (see Supplementary Table S9). Ponds with low pH and a wide macrophyte belt were dominated by poly-oxybiontic species, while in ponds with high pH or a narrow/missing macrophyte belt species with a moderate or low oxygen requirement were abundant (Fig. 5). The relative abundance of the two groups in these ponds was strongly negatively correlated (r = −0.935, p < 0.001); thus, only one of them could be dominant in each pond.

## Discussion

Although the pattern of changes varied considerably among traits, the overall picture was consistent with the SDH^23^, which predicts the dominance of environmental filtering under harsh conditions and the dominance of competition in more favourable habitats.

**Figure 6:**
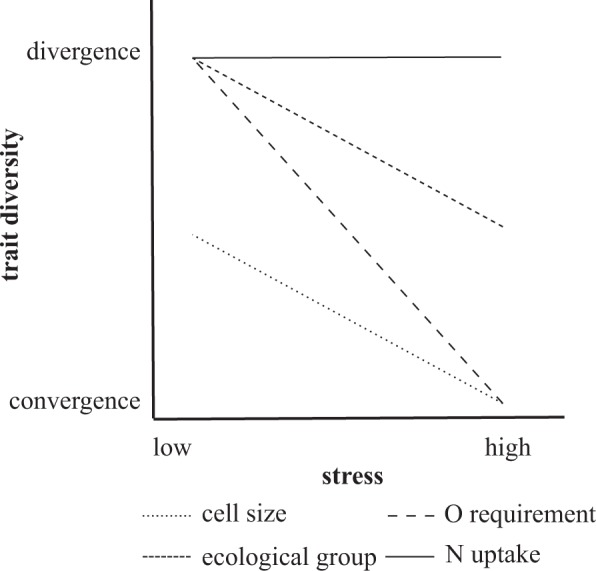
Summary of the observed changes in trait diversity along the stress gradient.

Salinity, pH and the width of the macrophyte belt were the three environmental variables that appeared as significant predictors of the convergence/divergence patterns. These variables were correlated, therefore we can interpret increasing salinity, increasing pH and decreasing width of macrophyte belts as indicators of increasing stress. This allows us to summarize the main trends of functional diversity along the stress gradient for all studied traits in a schematic figure (Fig. 6). The stress gradient defined by salinity, pH and width of macrophyte belt influences the composition of the diatom community, most probably through the availability of carbon dioxide and turbidity. If the pH is above 8.3, the amount of free carbon dioxide becomes very low, and practically only bicarbonate is present^42^. Thus diatoms inhabiting such ponds have to utilize other carbon sources^43^. The lack of a macrophyte belt results in the intensive resuspension of the particles by wind-induced turbulence, causing light limitation.

The expected response of the trait diversity (i.e. convergence or divergence) to stress depends on the ecological role of the trait^19,23^. According to the SDH, environmental filtering acts on traits that are important for tolerating the local environmental conditions, favouring convergence to optimal values and lowering trait diversity in stressful conditions, while favouring a random trait pattern in benign environments^19,23^. Cell size follows this expected pattern. The convergence is predominantly caused by the increasing dominance of micro-sized (100–300 μm^3^) diatoms (Fig. 3), whose reproduction and metabolic rate is high^44^. They can quickly utilize resources^45^, have an advantage in nutrient acquisition^46^ and effectively convert the nitrogen into biomass^44^, even at low carbon dioxide concentrations.

For traits related to resource use, the SDH predicts divergence in benign environments where resource competition plays a major role in forming the species composition, and random patterns in stressed environments, where the composition is shaped by habitat filtering^19,23^. The ecological group followed this expected pattern (Fig. 4). Although this is not a real resource use trait, it is probably associated with resource use strategy. The transition from divergence to neutrality corresponded to the increasing dominance of diatoms with EG3. Diatoms with EG3 were equivalent to diatoms of the motile guild in the system of Rimet & Bouchez^40^. A high proportion of the motile guild is frequently found in highly stressed environments^47^ like these ponds. Motility is a successful adaptation strategy in small aquatic environments where the algae are often exposed to harsh, adverse environmental conditions e.g. unfavourable light conditions^48^. Moving in the benthic mat, motile diatoms can avoid the lowered level of light and of suspended nutrients^49^ and can search for the most suitable habitat^50^.

Oxygen requirements changed from divergent to convergent along the gradient of increasing stress. This only partly supports the SDH, because it does not fit either expected pattern. At a high level of stress, TSS was high due to the lack of a wide macrophyte belt (see Supplementary Fig. S5), resulting in low light intensity, which limits the photosynthesis and oxygen production. Therefore, diatom species with a high oxygen requirement cannot exist there, and so species with a moderate or low oxygen requirement became dominant. In the most benign environment (low pH and a wide macrophyte belt), the oxygen requirement was divergent, in spite of the dominance of the poly-oxybiontic group (Fig. 5). Species with different oxygen requirements also use different carbon sources, thus oxygen requirement is a proxy of other traits related to resource use. Oxygen requirement is an example of traits that cannot be clearly classified into the two categories of resource use and stress tolerance traits. In stressed environments species with a high oxygen requirement are filtered out; therefore this trait behaves as a stress tolerance trait here. On the other hand, in benign environments, it behaves like a resource use trait, due to its correlation with other resource use traits.

Finally, the N-uptake strategy trait showed divergence in almost all ponds irrespective of environmental conditions. This is in accordance with the finding that limiting similarity causes divergence in traits related to resource use^15^. However, the relative abundance of N-uptake categories changed considerably. Sensitive N-autotrophic species could reach high relative abundance if the macrophyte belt was wide, and had a low relative abundance in other cases, especially if the pH was high (>8.37) (see Supplementary Fig. S7a). At high pH (>8.5), the bioavailable nitrogen concentration is low relative to the phosphorus concentration^51^, because of the intensive ammonia gas exhalation. The relative abundance of facultative N-heterotrophic species was low in all ponds, but significantly higher if the macrophyte belt was absent (see Supplementary Fig. S7b). The relative abundance of obligatory N-heterotrophic species was related to salinity: it was low if salinity was below 2.6 g L^−1^ (see Supplementary Fig. S7c), and high if salinity was higher. In ponds with higher salinity (>2.6 g L^−1^) and pH, nitrification is inhibited, so the denitrification increases nitrogen limitation^52^. This can be an explanation for the significant negative correlation between pH and the N:P ratio (see Supplementary Table S2). Moreover, in those ponds where the macrophyte belt was absent, diatoms could switch to heterotrophic metabolism as a response to low light intensity (TSS content is high in these ponds).

Until now, the SDH has only been tested in terrestrial communities, mainly in plant communities^17,18,26,27,53–56^, but Patric & Stevens^57^ have tested it in bat communities, and Kruk *et al*.^28^ in phytoplankton. Some studies support the SDH, while others have not found the expected pattern or have found the opposite.

According to our knowledge, this study is the first attempt to test the stress-dominance hypothesis in an aquatic environment. The most important conclusions of this study are the following:

- Cell size, a trait related only to stress tolerance, followed the SDH prediction: this is random in a benign environment and convergent in a stressed one.
- Ecological group, related to resource use, also followed the expected pattern of transition from divergence to a random pattern along the stress gradient
- Oxygen requirement, another limiting similarity, remained the dominant force forming a trait composition even in a strongly stressed environment, and thus showed divergence irrespective of the level of stress.
- Oxygen requirement cannot be obviously categorized into the two trait categories, since at low stress it behaves like a resource use trait, while at high stress it behaves as a stress tolerance trait; thus it shows patterns unpredicted by the SDH.

The controversial results in the literature could be caused by the fact that the stress gradient studied was too short, or that even the most extreme environment was not harsh enough, or that the studied trait was not responsive to the stress gradient studied. Moreover, it is also possible that the functional convergence/divergence along the stress gradient is idiosyncratic, i.e. that the SDH is valid only under specific circumstances. In such a situation, meta-analysis based on numerous case studies may help to uncover the regularities. Such meta-analysis should be based on many case studies covering a broad range of situations, including a broad range of habitat types. This study broadens the range of habitat types which could be included.

## Material and Methods

### Study area

The study area is situated in the northernmost part of the Kiskunság National Park (47°7.403′N 19°8.187′E), near the village of Apaj, in the plain of the Danube-Tisza Interfluve, Central Hungary, in an area of approximately 0.25 km^2^. The ponds developed in bomb craters can be characterized by varying areal extent, depth and salinity, conductivity, plant coverage and width of macrophyte belt. A more detailed description is presented in Vad *et al*.^33^.

### Sampling, water chemical analysis and microscopy investigations

Epiphytic diatom samples were taken from green common reed (*Phragmites australis*) stems, or, if it was absent, from alkali bulrush (*Bolboschoenus maritimus*) or narrowleaf cattail (*Typha angustifolia*) between 7 and 9 May 2014, following the recommendations of Bolla *et al*.^58^, in 48 ponds, resulting in a dataset which is heterogeneous in environmental factors (salinity, turbidity, pH, total phosphorus and nitrogen to phosphorus ratio; see Supplementary Table S1). A ten-centimetre section (or the maximal length in ponds with a depth of less than 10 cm) of the stems was cut, starting at 10 cm below the water surface. Stems were chosen randomly in five replicates per pond and treated as a composite sample.

In our study, we used those environmental variables which were significant drivers of community composition in a previous RDA analysis (not shown here). The water depth and diameter of each pond, along with the percentages of open water surface and macrophyte coverage (submerged and emergent plants were considered separately) were recorded *in situ*. Besides, we grouped the ponds into three categories based on the size of the surrounding macrophyte belt: wide (wider than approximately 30% of the diameter of the pond), narrow (narrower than approximately 30% of the diameter of the pond) and absent (cover not exceeding 10% and not forming a belt).

We used Eutech Cyber Scan PCD 650 (Eutech Instruments Europe B.V., Landsmeer, The Netherlands) field equipment for the *in situ* determination of pH and conductivity. The methods used to determine the concentration of total suspended solids (TSS), total phosphorus (TP), nitrate-nitrogen (NO_3_-N), ammonium-nitrogen (NH_4_-N) and chlorophyll *a* (Chl *a*) were described in Vad *et al*.^33^. In our study, we used dissolved inorganic nitrogen (DIN) data derived from the summarized concentrations of nitrate- and ammonium-nitrogen^33^. At least 400 valves were counted and identified to species level with an Olympus IX70 inverted microscope equipped with differential interference contrast (DIC) optics at a magnification of 1500× to estimate the relative abundance of each taxon in the sample. Further details of the microscopy investigation and diatom preparation methods are available in Vad *et al*.^33^.

### Trait data

We used four traits: ecological group related to ecological guilds^40,78^, cell size^41^, oxygen requirement^30^ and N-uptake strategy^30^.

Passy^30^ segregated diatom growth morphologies into three ecological guilds, based on their ability to use nutrient resources and their resistance to physical perturbation. The low profile guild contains diatoms of short stature attaching strongly to the surface. These diatoms can withstand resource limitation (nutrient, light) and disturbance (water current, grazers). Diatoms in the high profile guild have tall stature and attach weakly to the surface. They have superior competitive abilities in resource-rich but low disturbance habitats. The motile guild encompasses species displaying active, regulated movement within the benthic habitat. Movement is coupled to mucilage secretion and substratum adhesion along the slit in the cell wall (raphe) characteristic of motile species^30^. Members of the motile guild are physically capable of selecting the most suitable habitat and avoiding stress by moving in the benthic mat. This latter guild comprises mostly eutrophic and pollution tolerant species and they are superior competitors for nutrients in nutrient-rich environments^30^.

The guild classification proposed by Passy^30^ and modified by Rimet & Bouchez^40^ was originally developed for rivers, but it is also applicable in lentic habitats^59^. Because the guild has another meaning in animal ecology^39^, we use the term ecological group (as proposed by Tapolczai *et al*.^39^).

The ecological group can reflect the trade-off between stress tolerance and competitive dominance. It can be hypothesized that in stressful conditions diatoms with an EG1 trait (corresponding to the low profile guild) become dominant, while in more favourable environments diatoms with superior competitive capabilities (trait categories EG2 and EG3, equivalent to high profile and motile guilds, respectively) become more abundant. The relative abundance of EG4 (corresponding to the planktic guild) is low in small water bodies, because of the absence of phytoplankton.

According to the allometric theory^60^, cell size is the major determinant of the specific physiological activities of algae^6^ such as growth, nutrient uptake and light capture. Small cells can be more efficient in utilizing low light or limited nutrients, and thus become dominant in more stressful environments^61^. In contrast, in more favourable conditions large cells have superior competitive abilities. Accordingly, trait diversity would be low at both ends of the stress gradient. The correlation of the mean relative surface areas and surface/volume ratio (S/V) with abiotic factors were very similar to the mean biovolumes of diatoms^62^. Stenger-Kovács *et al*.^59^ used the length/width ratio (L/W) beside the biovolume as a morphological trait, but salinity can significantly affect the valve morphology; however the effects can be different in the different species: length and width or only width can be significantly affected^63^, so we do not consider using this trait to be a good decision.

Oxygen is produced by photosynthesis, which is influenced by light, temperature and nutrient levels. In light-limited habitats (such as in soda ponds where the content of suspended solids is high) diatoms may rely on heterotrophic metabolism^64^ shifting from oxygen producers to oxygen consumers. Van Dam *et al*.^30^ established a classification of the oxygen requirements of diatoms based on the minimum oxygen concentration of waters that species need. The oxygen requirement trait is related to stress tolerance. In a habitat that is stressful due to a low oxygen concentration, diatoms with a low oxygen requirement will be dominant and species with a high oxygen requirement will be missing; therefore, the diversity of this trait will be low. Conversely, under favourable conditions diatoms requiring a higher oxygen concentration could also become abundant, resulting in an increase in trait diversity.

Several studies have shown that diatoms are able to assimilate organic nitrogen compounds e.g.^65^. Heterotrophic nitrogen-uptake serves as an additional source of nitrogen for diatoms, particularly under nitrogen-poor conditions, which may occur in soda waters^66^. Diatoms with this ability are nitrogen-heterotrophic, while diatoms that can utilize only inorganic nitrogen are nitrogen-autotrophic^30^. In stressful environments, the ability of heterotrophic nitrogen-uptake is advantageous, allowing nitrogen-heterotrophic diatoms to be dominant. In favourable conditions, nitrogen-autotrophic taxa could be successful. Thus, trait diversity would be low in a stressful environment, but high in a benign one.

We used the term ‘ecological group’ based on the system proposed by Rimet & Bouchez^40^ (the use of the term ‘guild’ was changed according to the Tapolczai *et al*.^40^, as we mentioned above). For cell size, the categories were those determined by Lange, Townsend, & Matthaei^41^, for oxygen requirement and nitrogen uptake strategy the classifications of van Dam *et al*.^30^ were applied. The values of these traits were obtained from the OMNIDIA 6.0.2 database^67^.

### Statistical analysis

If traits vary independently among species, observed patterns for each trait can be interpreted separately. On the other hand, in pairs of correlated traits, effects forming the distribution of a trait can also indirectly affect another through the correlation between them. Associations between traits were tested by Chi-square statistics. Since the assumption that binomial distribution can be approximated by normal distribution was not satisfied for some rare combinations, p-values were calculated by a randomization test. If the Chi-square statistic showed a significant association, Freeman-Tukey deviates were calculated to explore trait combinations which were significantly more/less frequent than expected. Correlations between environmental variables were tested using Spearman’s rank correlation.

Dissimilarity matrices of species were calculated for each trait separately, applying Gower distances which can handle ordered (cell size, N uptake, oxygen requirement) and unordered (ecological group) categorical data^68,69^, using Eq. 2a and 2b in Podani^68^ for ordered traits. Functional diversity was calculated from these dissimilarity matrices and species’ relative abundances, applying Rao’s quadratic entropy^70^. Instead of separately testing trait convergence due to environmental filtering and trait divergence due to competition, we needed a procedure that can measure the strength of both processes. Therefore, during randomization trait values were reshuffled among species^15^. Botta-Dukát & Czúcz^12^ referred to this type of randomization as between plot randomization. Their simulation studies proved that this test can detect the existing trait convergence due to environmental filtering if the dataset covers a wide range of environmental variables, and can also detect the existing divergence due to competition if there is no direct or indirect effect of environmental filtering on that trait in that environment^12^. Probabilities of type I error (p-values) were estimated for each plot separately from 9999 random communities.

Since the distribution of Rao’s quadratic entropy in the randomized communities were often highly right-skewed^18^, standardized effect sizes^71^ are inappropriate to measure the strength of convergence/divergence^11,72^. Therefore we followed the approach suggested by Botta-Dukát^73^ and used probit-transformed p-values, (hereafter called effect size) as dependent variables in the subsequent analyses. Probit-transformation transforms probabilities into a minus infinity to infinity range^74^. If the null-distribution is Gaussian, the calculated effect size is asymptotically equal to Gotelli’s standardized effect size (SES)^71^, and if the null-hypothesis is true, the effect size follows standard normal distribution even when the null-distribution is highly skewed. To reveal the overall (i.e. not regarding position along environmental gradients) convergence/divergence pattern, the departure of the mean effect size from zero was tested by a one-sample t-test.

To describe trait composition, Ricotta & Moretti^75^ recommended quantifying both the mean and the dispersion of the trait values. While Rao’s quadratic entropy quantifies dispersion^76^, for categorical traits the community weighted mean (CWM) can be calculated for binary dummy variables instead of the categorical variables themselves, resulting in the relative abundance of each category.

Due to the large number of potentially important environmental variables (13 variables), the relationships between them and the effect size values were explored by fitting conditional inference-based decision trees. Decision trees (also known as classification and regression trees^77^) are non-parametric statistical methods that can handle nonlinear relationships and mixed type (i.e. both categorical and continuous) independent variables, even if there is multicollinearity among them, and their results are easy to interpret^78^. The selected algorithm offers unbiased variable selection and a statistically sound stopping rule using p-values corrected for the effect of simultaneous hypothesis testing^79^, which eliminates the variable selection bias and problems of under- and over-fitting. The relative abundance of trait categories (i.e. CWM) in groups resulting from decision trees were compared by Wilcoxon (for two groups) or Kruskal-Wallis tests (for more than two groups). Note that relative abundances are summed to unity, therefore tests are not independent.

All analyses were done in an R 3.1.1 environment^80^ using ‘party’^80^ and ‘FD’^81^ add-on packages.

## Supplementary information

Supplementary Info

## Data Availability

Raw data are archived in Harvard Dataverse (10.7910/DVN/FGXN5V, available at 10.7910/DVN/FGXN5V).
