# Supplementary material for: Trait-based community assembly of epiphytic diatoms in saline astatic ponds: a test of the stress-dominance hypothesis
Source: Sci Rep. 2019 Oct 31;9:15749. doi: 10.1038/s41598-019-52304-4 (PMC6823390; doi:10.1038/s41598-019-52304-4)
Supplement: Supplementary file 1 — Supplementary Info [file 41598_2019_52304_MOESM1_ESM.docx]

**Supporting information for Trait-based community assembly of epiphytic diatoms in saline astatic ponds: a test of the stress-dominance hypothesis**

Éva Ács, Angéla Földi, Csaba Ferenc Vad, Zsuzsa Trábert, Keve Tihamér Kiss, Mónika Duleba, Gábor Borics, István Grigorszky, Zoltán Botta-Dukát,

**Tables**

Table S1. Ranges, mean and median values of the environmental variables recorded for the 48 investigated bomb crater ponds.

|  | **min** | **max** | **mean ± SE** | **Median** |
| --- | --- | --- | --- | --- |
| Area (m^2^) | 7.1 | 86.5 | 41.2 ± 2.7 | 38.5 |
| Depth (cm) | 4.0 | 60.0 | 36.8 ± 1.9 | 37.5 |
| Salinity (g L^-1^) | 0.97 | 5.5 | 2.61±0.1 | 2.64 |
| Conductivity (mS cm^-1^) | 1.3 | 5.1 | 3.4 ± 0.2 | 3.4 |
| pH | 7.8 | 9.0 | 8.4 ± 0.0 | 8.4 |
| Secchi-depth (cm) | 4.0 | 50.0 | 18.5 ± 1.5 | 18.5 |
| Turbidity (NTU) | 4.8 | 691.0 | 116.5 ± 23.4 | 56.5 |
| Total suspended solids (mg L^-1^) | 6.8 | 388.0 | 77.2 ± 12.7 | 46.5 |
| Total phosphorus (μg L^-1^) | 32.7 | 1693.6 | 271.6 ± 46.5 | 126.6 |
| Chlorophyll *a* (µg L^-1^) | 0.0 | 387.6 | 26.8 ± 9.2 | 10.1 |
| Nitrate nitrogen (mg L^-1^) | 0.1 | 0.8 | 0.3 ± 0.0 | 0.3 |
| Ammonium nitrogen (mg L^-1^) | 0.01 | 3.51 | 0.30 ± 0.09 | 0.13 |
| Open water surface (%) | 40.0 | 99.5 | 85.4 ± 2.2 | 90.0 |
| Submerged macrophyte coverage (%) | 0.0 | 20.0 | 0.8 ± 0.5 | 0.0 |
| Emergent macrophyte coverage (%) | 0.5 | 60.0 | 13.6 ± 2.2 | 6.0 |

Table S2. Spearman's rank correlations between continuous environmental variables. Correlations significant at 1% level are typed by bold.

|  | salinity | N:P ratio | TSS | area | Z_depth | pH | DIN | TP | Chl_a |
| --- | --- | --- | --- | --- | --- | --- | --- | --- | --- |
| N:P ratio | **-0.4810** |  |  |  |  |  |  |  |  |
| TSS | **0.5727** | **-0.5872** |  |  |  |  |  |  |  |
| area | **0.4881** | -0.2981 | 0.1682 |  |  |  |  |  |  |
| Z_depth | 0.1441 | 0.2260 | -0.3408 | **0.4010** |  |  |  |  |  |
| pH | **0.7417** | **-0.4234** | **0.5893** | 0.3363 | 0.0283 |  |  |  |  |
| DIN | 0.3079 | 0.2148 | 0.1810 | -0.1469 | 0.0180 | 0.3482 |  |  |  |
| TP | **0.6762** | **-0.7923** | **0.7604** | 0.2168 | -0.3109 | **0.6413** | 0.3320 |  |  |
| Chl_a | -0.0584 | -0.2367 | 0.3038 | -0.1231 | **-0.5610** | 0.0182 | -0.1243 | 0.2898 |  |
| Ca | 0.0372 | 0.2503 | -0.2749 | -0.1596 | **0.4413** | -0.1298 | 0.3598 | -0.1566 | -0.2539 |

Table S3. Cross-classification of diatom species by cell size and ecological groups. The two traits are statistically independent (Chi-squared = 17.169, p-value = 0.1242)

|  |  | **Cell size** | | | | |  |
| --- | --- | --- | --- | --- | --- | --- | --- |
|  |  | nano | micro | mezo | macro | very large | Σ |
| **Ecological group** | EG1 | 2 | 11 | 5 | 9 | 21 | 48 |
|  | EG2 | 0 | 3 | 7 | 4 | 3 | 17 |
|  | EG3 | 1 | 2 | 3 | 0 | 2 | 8 |
|  | EG4 | 0 | 0 | 1 | 0 | 0 | 1 |
|  | Σ | 3 | 16 | 16 | 13 | 26 | 74 |

Table S4. Cross-classification of diatom species by cell size and oxygen requirement traits. The two traits are not statistically independent (Chi-squared = 22.795, p-value = 0.0254). Significant departures - based on Freeman-Tukey deviates - are marked by bold. Blue characters mean lower, red characters mean higher observed frequency than expected (shown in brackets) if the two classification is fully independent.

|  |  | **Cell size** | | | | |  |
| --- | --- | --- | --- | --- | --- | --- | --- |
|  |  | nano | micro | mezo | macro | very large | Σ |
| **Oxygen requirement** | 1 | 1 (0.49) | **6 (2.59)** | 3 | 2 | **0 (4.22)** | 12 |
|  | 2 | 1 | **1 (4.76)** | 4 | **8 (3.86)** | 8 | 30 |
|  | 3 | 0 | 6 | 4 | 1 | 11 | 22 |
|  | 4 | 1 | 3 | 5 | 2 | 7 | 18 |
|  | Σ | 3 | 16 | 16 | 13 | 26 | 74 |

Table S5. Cross-classification of diatom species by cell size and nitrogen uptake traits. The two traits are not statistically independent (Chi-squared = 29.097, p-value = 0.0036). Significant departures - based on Freeman-Tukey deviates - are marked by bold. Blue characters mean lower, red characters mean higher observed frequency than expected (shown in brackets) if the two classification is fully independent.

|  |  | **Cell size** | | | | |  |
| --- | --- | --- | --- | --- | --- | --- | --- |
|  |  | nano | micro | mezo | macro | very large | Σ |
| **N uptake** | sensitive N-autotrophic | 0 | 5 | 5 | 3 | 5 | 18 |
|  | tolerant N-autotrophic | 1 | 5 | 7 | 10 | 20 | 43 |
|  | facultative N-heterotrophic | **2 (0.28)** | 2 | 2 | 0 | 1 | 7 |
|  | obligate N-heterotrophic | 0 | **4 (1.30)** | 2 | 0 | **0 (2.11)** | 6 |
|  | Σ | 3 | 16 | 16 | 13 | 26 | 74 |

Table S6. Cross-classification of diatom species by ecological group and oxygen requirement traits. The two traits are not statistically independent (Chi-squared = 22.811, p-value = 0.0023). Significant departures - based on Freeman-Tukey deviates - are marked by bold. Blue characters mean lower, red characters mean higher observed frequency than expected (shown in brackets) if the two classification is fully independent.

|  |  | **Ecological group** | | | | Σ |
| --- | --- | --- | --- | --- | --- | --- |
|  |  | EG1 | EG2 | EG3 | EG4 |  |
| **Oxygen requirement** | polyoxybiont | **3 (7.78)** | **8 (2.76)** | 1 | 0 | 12 |
|  | oxybiont | 16 | 4 | 1 | 1 | 22 |
|  | Moderate | 14 | 3 | 5 | 0 | 22 |
|  | Low | 15 | 2 | 1 | 0 | 18 |
|  | Σ | 48 | 17 | 8 | 1 | 74 |

Table S7. Cross-classification of diatom species by ecological group and nitrogen uptake traits. The two traits are statistically independent (Chi-squared = 15.654, p-value = 0.1182).

|  |  | **Ecological group** | | | |  |
| --- | --- | --- | --- | --- | --- | --- |
|  |  | EG1 | EG2 | EG3 | EG4 | Σ |
| **N uptake** | sensitive N-autotrophic | 10 | 8 | 0 | 0 | 18 |
|  | tolerant N-autotrophic | 28 | 6 | 8 | 1 | 45 |
|  | facultative N-heterotrophic | 4 | 3 | 0 | 0 | 7 |
|  | obligate N-heterotrophic | 6 | 0 | 0 | 0 | 6 |
|  | Σ | 48 | 17 | 8 | 1 | 74 |

Table S8. Cross-classification of diatom species by oxygen requirement and nitrogen uptake traits. The two traits are not statistically independent (Chi-squared = 38.173, p-value = 0.0001). Significant departures - based on Freeman-Tukey deviates - are marked by bold. Blue characters mean lower, red characters mean higher observed frequency than expected (shown in brackets) if the two classification is fully independent.

|  |  | **Oxygen requirement** | | | |  |
| --- | --- | --- | --- | --- | --- | --- |
|  |  | polyoxybiont | oxybiont | moderate | low | Σ |
| **N uptake** | sensitive N-autotrophic | **10 (2.92)** | 6 | **1 (5.35)** | **1 (4.38)** | 18 |
|  | tolerant N-autotrophic | **2 (6.97)** | 15 | 16 | 10 | 45 |
|  | facultative N-heterotrophic | 0 | 1 | 2 | 4 | 7 |
|  | obligate N-heterotrophic | 0 | **0 (1.78)** | 3 | 3 | 6 |
|  | Σ | 12 | 22 | 22 | 18 | 74 |

Table S9. Minimum, median and maximum values of the relative abundance of the trait categories.

| Trait | Categories | Minimum | Median | Maximum |
| --- | --- | --- | --- | --- |
|  |  | relative abundance of categories | | |
| Ecological group | 1: EG1 | 0.002 | 0.130 | 0.640 |
|  | 2: EG2 | 0 | 0.141 | 0.679 |
|  | 3: EG3 | 0.097 | 0.689 | 0.948 |
|  | 4: EG4 | 0 | 0 | 0.002 |
| Cell size | 1: nano (<100 μm^3^) | 0 | 0.017 | 0.232 |
|  | 2: micro (100 ≤ 300 μm^3^) | 0.092 | 0.673 | 0.995 |
|  | 3: meso (300 ≤ 600 μm^3^) | 0.005 | 0.172 | 0.612 |
|  | 4: macro (600 ≤ 1500 μm^3^) | 0 | 0.025 | 0.214 |
|  | 5: very large (>1500 μm^3^) | 0 | 0.012 | 0.052 |
| Oxygen requirement | 1: polyoxybiontic (saturation: 100%) | 0 | 0.116 | 0.903 |
|  | 2: oxybiontic (75%) | 0 | 0.042 | 0.166 |
|  | 3: moderate (50%) | 0.031 | 0.356 | 0.955 |
|  | 4: low (30%) | 0.039 | 0.257 | 0.752 |
|  | 5: very low (10%) | Species belonging to this category do not occur in the samples | | |
| N-uptake | 1: sensitive N-autotrophic | 0 | 0.101 | 0.635 |
|  | 2: tolerant N-autotrophic | 0.171 | 0.574 | 0.931 |
|  | 3: facultative N-heterotrophic | 0 | 0.005 | 0.061 |
|  | 4: obligatory N-heterotrophic | 0.014 | 0.169 | 0.639 |

**Figures**

Figure S1. Relationship between salinity and pH in the studied ponds. Each point represents one pond, while the line is the prediction of the fitted linear regression.

|  |  |
| --- | --- |
| **a**) | **b**) |

Figure S2. Box-plots of salinity (a) and pH (b) in the three categories of macrophyte belt width.

Figure S3. Relationship between salinity and species richness in the studied ponds.

|  |  |
| --- | --- |
| **a**) | **b**) |
|  |  |
| **c**) | **d**) |
|  |  |
| **e**) | **f**) |

Figure S4. Boxplots of environmental conditions of ponds belonging to categories created by conditional inference tree fitted in effect size calculated for cell size trait.

|  |  |
| --- | --- |
| **g**) | **h**) |
|  |  |
| **i**) | **j**) |
|  |  |
| **k**) | **l**) |

Figure S4. (continued) Boxplots of environmental conditions of ponds belonging to categories created by conditional inference tree fitted in effect size calculated for cell size trait.

|  |  |
| --- | --- |
| **a**) | **b**) |
|  |  |
| **c**) | **d**) |
|  |  |
| **e**) | **f**) |

Figure S5. Boxplots of environmental conditions of ponds belonging to categories created by conditional inference tree fitted in effect size calculated for oxygen requirement trait.

|  |  |
| --- | --- |
| **g**) | **h**) |
|  |  |
| **i**) | **j**) |
|  |  |
| **k**) | **l**) |

Figure S5. (continued) Boxplots of environmental conditions of ponds belonging to categories created by conditional inference tree fitted in effect size calculated for oxygen requirement trait.

|  |  |
| --- | --- |
| a) | **b**) |
|  |  |
| c) | **d**) |
|  |  |
| **e**) | **f**) |

Figure S6. Boxplots of environmental conditions of ponds belonging to categories created by conditional inference tree fitted in effect size calculated for combined trait.

|  |  |
| --- | --- |
| **g**) | **h**) |
|  |  |
| **i**) | **j**) |
|  |  |
| **k**) | **l**) |

Figure S6. (continued) Boxplots of environmental conditions of ponds belonging to categories created by conditional inference tree fitted in effect size calculated for combined trait.


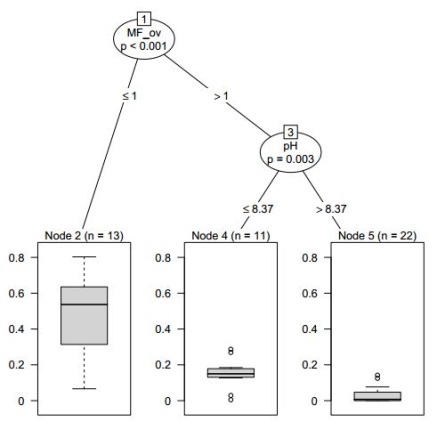

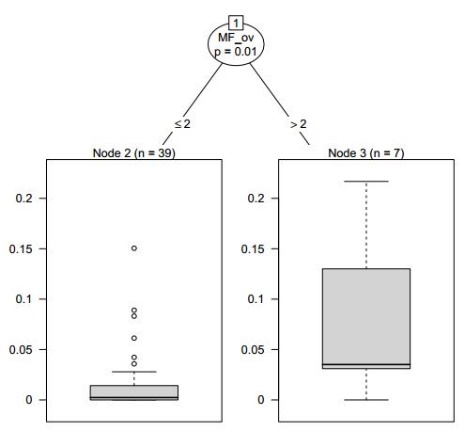

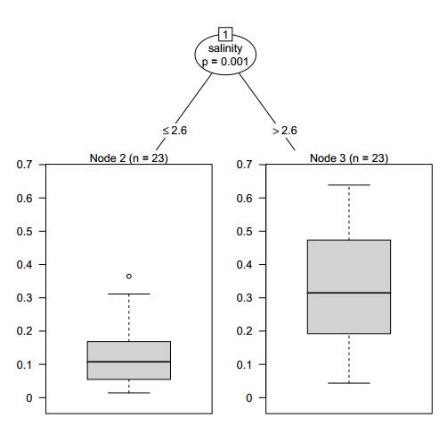


1. **b**) **c**)

Figure S7. Regression tree model of the dependence of relative abundace of N-uptake strategies on environmental conditions. a) sensitive N-autotrophic species, b) facultative N-heterotrophic species, c) obligatory N-heterotrophic species.
